# Pathogenesis of Retraction Pocket of the Tympanic Membrane—A Narrative Review

**DOI:** 10.3390/medicina57050425

**Published:** 2021-04-28

**Authors:** Milan Urík, Miroslav Tedla, Pavel Hurník

**Affiliations:** 1 Department of Paediatric Otorhinolaryngology, Faculty of Medicine of Masaryk University in Brno, University Hospital Brno, 61300 Brno, Czech Republic; urik.milan@fnbrno.cz; 2 Department of Otorhinolaryngology, Head and Neck Surgery, Comenius University, University Hospital, 85107 Bratislava, Slovakia; miro.tedla@gmail.com; 3 Institute of Pathology, University Hospital Ostrava, 70852 Ostrava, Czech Republic; 4 Institute of Animal Physiology and Genetics, Czech Academy of Sciences, 60200 Brno, Czech Republic

**Keywords:** retraction pocket, tympanic membrane, pathogenesis, pathology, chronic otitis media, cholesteatoma

## Abstract

Several theories describe the development of the retraction pocket of the tympanic membrane (RP). Many authors suggest that the negative middle ear pressure is the main reason responsible for developing this condition. A narrative review has been undertaken, and conclusions are drawn reflecting a current knowledge with our new observations in the histological and immunohistochemical study. Recent studies show the important role of inflammation in the development and progression of RP. A review of the available literature shows that the inflammation plays a key role in pathogenesis of the RP and its progression to the cholesteatoma. We support this statement with our new results from histological and immunohistochemical analysis of the RPs.

## 1. Introduction

**Figure 1:**
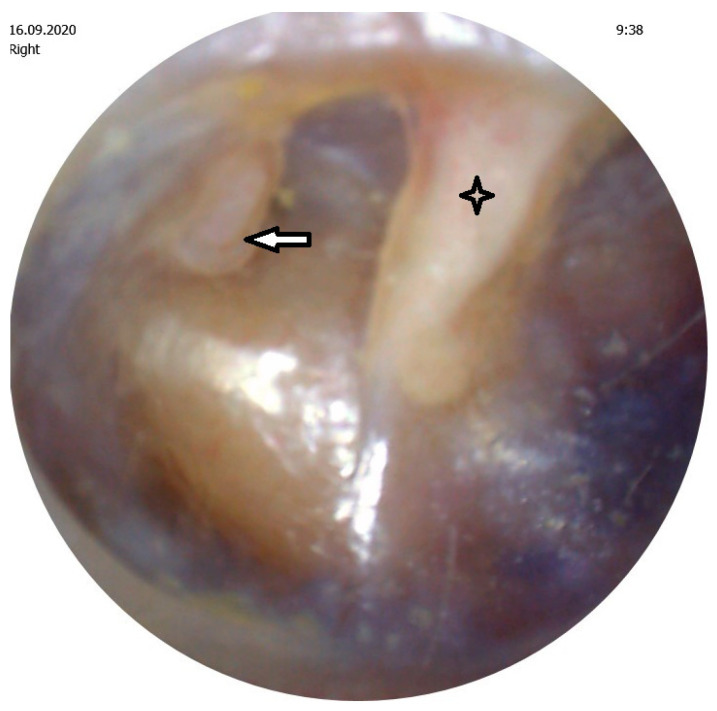
Retraction pocket of pars tensa caused erosion of the incudostapedial joint. 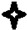 Malleus, 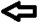 Erosion of the incudostapedial joint.

Retraction pocket (RP) of the tympanic membrane is a common entity, especially in children. The danger of RP lies in ossicular chain erosion (Figure 1), cholesteatoma formation and potentially life-threatening complications of cholesteatoma. No consensus exists about the optimal treatment. We have a lot of new knowledge regarding pathogenesis, but there are still many unknown factors that influence development of this condition. 

Epidemiological studies have shown that the retraction pocket is a relatively rare disease and it largely affects the child population. In 1994, Stangerup published a study where it examined 294 healthy children aged 5 to 16 years and found the occurrence of retraction pockets in 26% in the area pars flaccida and at 3.7% in the pars tensa area [1]. The largest long-term study in a healthy population includes 7000 children under the age of 10, when the prevalence of retraction pockets in the pars flaccida was 9.6 % and pars tensa 7.9%. Most retraction pockets were classified as “mild”, only a small part represented serious cases. This study also showed frequent bilateral disability retraction pocket [2]. Relatively frequent occurrence of retraction pockets in the younger age group of children up to 10 years was not found in the older age group. A study of children aged 8–18 years demonstrated that a significant proportion of tympanic pathologies found on first examination healed spontaneously during a 10-year follow-up [3].

Histologically the normal tympanic membrane consists of an external epithelial layer, middle layer, and internal mucosal layer. A healthy tympanic membrane is relatively tough and elastic, this is due to the structure and properties of its middle layer. 

The external layer is formed by the epidermis, which has a protective function and is composed of stratified keratinizing epithelium. Keratinizing cells from the surface gradually migrate from the center of the tympanic membrane to the periphery and further outwards from the ear canal. This ensures self-cleaning ability [4]. The middle layer is formed by connective tissue with a predominance of collagen fibers arranged in two layers (outer radial and inner circular); elastic fibers also occur [5]. The thickness of this layer in the pars tensa region ranges from 0.04 mm in the posterosuperior quadrant up to 0.09 mm in the annulus region. It is mostly collagen type II and IV, which typically occurs in the cartilage. Collagen fibers with admixture of fibers elastic determine the basic mechanical properties of the drum. The middle layer of the eardrum also contains the delicate vascular and nerve plexus [6]. The inner layer (lamina interna membranae tympani) of the eardrum is formed by the middle ear cavity mucosa and its function is protection, metabolism and nutrition of the middle layer of the tympanic membrane. 

The three most used classification systems of retraction pockets are Sadé [7] (for pars tensa RPs), Tos [8] (for pars flaccida RPs), and Charachon [9]. These systems grade the severity of RP, but they do not explain the pathogenesis or possibility of progression of retraction pocket. The association of severity of the retraction pocket in the development of acquired cholesteatoma is the key point, however, there is no consensus existing staging systems on retraction pockets or cholesteatoma in serving this purpose [10]. 

Retraction pocket is a localized area of the tympanic membrane invaginated into the tympanic cavity. It is much more common in children than in adults. The incidence of atrophy increases from 4% at age 4 years to 11% at age 16 years [11]. A normal tympanic membrane is tough and elastic, but the retraction pocket is flexible and collapses into the tympanic cavity. The place of weakness is most often located near the anatomical structures such as incisura Rivini, scutum or malleus [12]. It can affect both parts of the TM, pars tensa or pars flaccida. Many theories explain the pathomechanism of the development of the retraction pocket. 

## 2. The Eustachian Tube and Pathogenesis of the Retraction Pocket

One of the theories identifies the long-term negative middle ear pressure caused by dysfunction of the Eustachian tube as a main reason causing development of the retraction pocket. Normal TM has a certain capacity to buffer the pressure changes [13]. Ears that have anomalies in the volume and ventilation of the epitympanum may be more susceptible to retraction pockets [14]. The healthy Eustachian tube equilibrates the pressure between middle ear and nasopharynx. There are a lot of factors causing dysfunction of the Eustachian tube (congenital malformations, mucosal oedema, adenoid vegetations and many others). This ex vacuo theory is based on clinical studies and animal experiments [15,16,17], but the results are contradictory. In many children after adenoidectomy or ventilation tube insertion, retraction pocket or cholesteatoma [18,19] developed. RP can occur even in the absence of negative pressure in the middle ear while the ventilation tube is inserted in the TM. It is probably caused by the presence of chronic inflammatory changes in the musculus tensor tympani, which retracts malleus [20]. Some studies point out a fact that negative middle ear pressure alone is not sufficient to cause retraction [21,22,23,24]. In conclusion, there is no clear evidence that negative middle ear pressure alone will cause invagination of normal tympanic membrane.

## 3. The Relationship of Otitis Media and Pathogenesis of the Retraction Pocket

It is true that the retraction pockets are commonly observed in children with recurrent acute otitis media or otitis media with effusion. Sadé described that inflammation as responsible for the degeneration of the lamina propria of the tympanic membrane [6]. Experimental study showed that the initial reason for the development of cholesteatoma from the retraction pocket is local inflammation of the mucosa of the middle ear [25]. A wide spectrum of theories describes the pathogenesis of acquired cholesteatoma [26] and tries to clarify why keratinizing squamous epithelium starts growing from the outer layer of the tympanic membrane into the cavity of the middle ear (metaplasia theory, migration theory, papillary ingrowth theory). However, there is no clear evidence why the epithelial cells prefer to migrate medially rather than laterally as is normal for cleaning of the healthy tympanic membrane. Of course, some common middle ear pathogens increase proinflammatory cytokines in monocytes, especially intracellular adhesion molecule–1(ICAM-1) which mediate to adhesion of leukocytes to the vascular epithelium and the transition to the extravascular space by the proinflammatory signaling, and soluble forms increase in the middle ear effusion and serum due to autogenic infections. ICAM-1 was significantly higher in patients with cholesteatoma, ossicular chain defects and tympanic membrane retraction [27].

In 2018, Hüttenbrink published the self-healing hypothesis as the starting point of a tympanic membrane retraction [28]. This idea interprets the horizontal migration of skin into the middle ear cavity as a self-healing process, repairing an underlying inflammation in the tympanic cavity, through the overgrowth and contact with immunologically active tissue. This theory is supported by analogous phenomena that exist, e.g., the migration of the omentum towards a local inflammation in the abdomen [29]. The findings in 209 second look surgeries (from authors) provide the first explanation of the origin of retraction pockets that is compatible with the various characteristics of original or recurrent cholesteatoma. The process of changes in the TM structure may evolve to the cholesteatoma formation [30]. 

## 4. The Role of Inflammation in Pathogenesis of the Retraction Pocket

**Figure 2:**
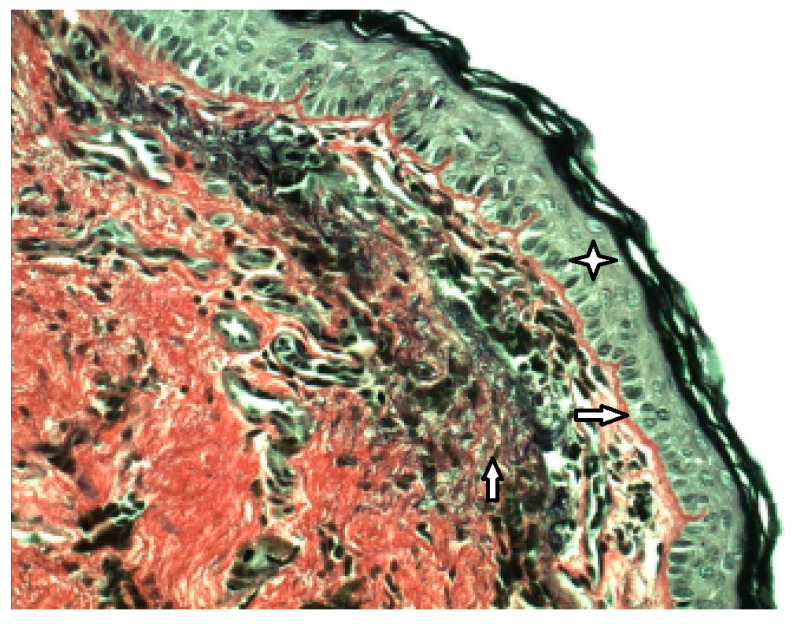
Fragmentation of the elastic fibers in the middle layer of tympanic membrane, histology of retraction pocket, Verhoeff, (400×). 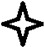 External epithelial layer, 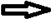 Basal membrane, 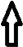 Fragmented elastic fibers in the middle layer.

**Figure 3:**
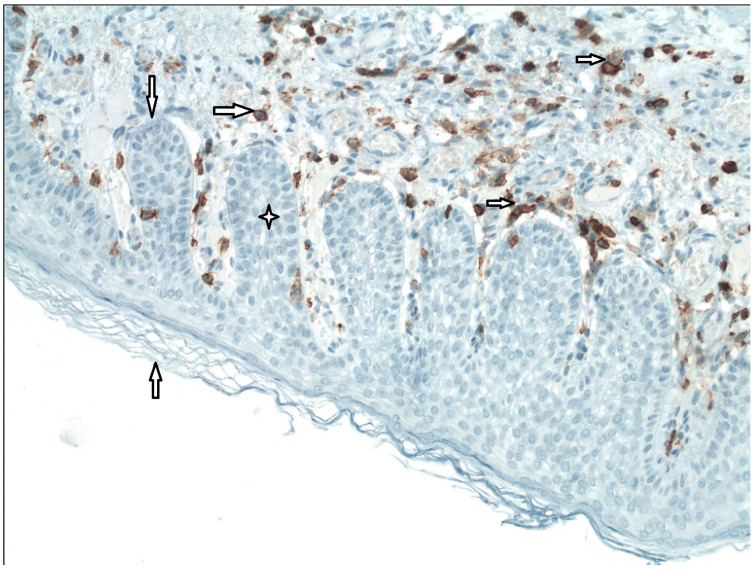
Immunohistochemistry–CD45 LCA, inflammation (200×). 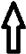 External epithelial layer, 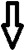 Basal membrane, 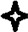 Rete peg (area of ingrowing squamous epithelium), 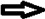 White blood cells of all categories (CD45 LCA+).

**Figure 4:**
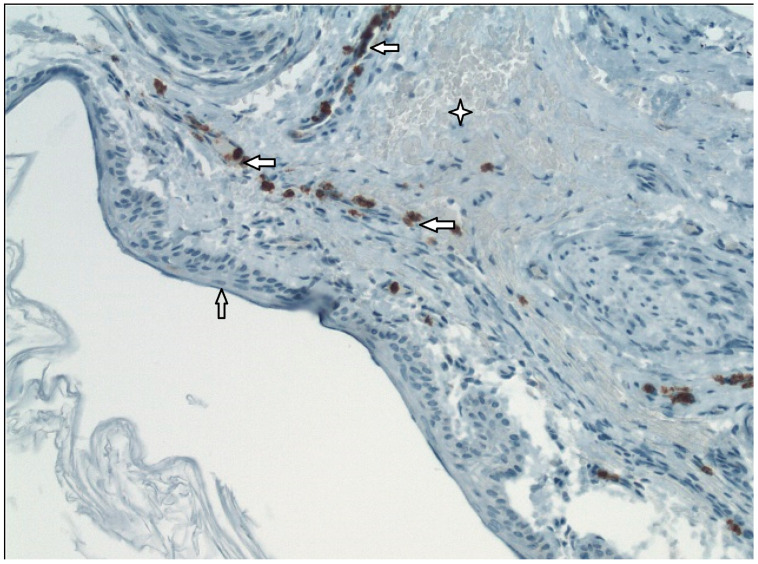
Immunohistochemistry–MMP9 in the middle layer of tympanic membrane (200×). 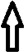 External epithelial layer, 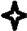 Middle layer of the tympanic membrane, 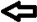 MMP9+.

We conducted the histological and immunohistochemical analysis of 31 pars tensa retraction pockets (stadium II and III by Charachon) in children. We identified the pathological anomalies in the structure of external and middle layers of the tympanic membrane compared with normal healthy tympanic membrane. We identified subepithelial inflammation infiltrate in 86% of patients, disruption of a double layer of collagen fibers and its infiltration by neutral mucosubstances in 87% and fragmented elastic fibers in 96% (Figure 2) [31]. Degeneration of the middle collagenous fibrous layer caused by inflammation was observed by Sade [1] or Yoon [32]. We also described hyperkeratosis (100%) and ingrowing rete pegs (71%) [31]. In the outer layer of the tympanic membrane (epidermis), we identified a presence of Ki67 in the cells of both basal and suprabasal layers. These observations suggest an increase of proliferation activity in squamous epithelia. The process of keratinocyte proliferation within cholesteatoma epithelium is important not only for understanding the complex mechanisms of the pathogenesis of this disease, but also to anticipate the possible development or recurrence of cholesteatoma [30,31,32,33]. We identified CD45 LCA positivity, which is typical for all categories of white blood cells, in both the tympanic membrane’s external epithelial layer and its central connective tissue layer (Figure 3). We observed MMP9 positivity in the central tympanic membrane layer, thereby indicating a presence of degenerative processes in the extracellular matrix of connective tissue (Figure 4) [34]. MMP affect both inhibitory and stimulating effect on cell apoptosis [35]. Our observations show that it is inflammation that plays a key role in the pathogenesis of the retraction pocket. This statement is supported by other studies [26,36,37,38]. Of course, these anomalies commonly occur in the matrix and perimatrix of cholesteatoma, so our observations support the retraction theory of development of cholesteatoma.

Degeneration of the lamina propria of the tympanic membrane is a long-term process. Inflammation leads to destruction of the collagen and elastic fibers. Mediators of inflammation lead to the release of collagenases and destroy the disulfate bridges. For this reason, the tympanic membrane becomes thin and elastic properties are changed. Weakened tympanic membrane loses its resistance to pressure changes. After the initial insult and loss of strength, inflammation is no longer necessary for progression of the retraction pocket. The problem of retraction pockets lies in the loss of original histological and anatomical structure, therefore its inability to resist the negative middle ear pressure.

Based on this knowledge, it is very important to prevent and treat the inflammation early. All children with recurrent acute otitis media or secretory otitis media should have a comprehensive examination by a pediatrician and otorhinolaryngologist. We can perform an adenoidectomy, insertion of the ventilation tube to the tympanic membrane or a special treatment in case of immunodeficiency. Watch and wait strategy can be dangerous and it can lead to damage of the middle ear ossicles or to development of cholesteatoma in many cases.

## 5. Conclusions

All of these claims also support the basic mechanism of why an intact tympanic membrane invaginates into the tympanic cavity and also support the retraction theory of cholesteatoma. The first step in pathogenesis of the retraction pocket is affection of the inflammation from the middle ear mucosa to the tympanic membrane. Inflammation caused the pathological changes in the middle layer of the tympanic membrane: hypervascularisation, degradation of the double layer of collagen fibers, fragmentation of the elastic fibers and others. These changes lead to significant weakening of the tympanic membrane, especially in pars tensa. This area of the tympanic membrane is more flexible and less resistant to negative pressure in the middle ear in children with dysfunction of the Eustachian tube. Inflammation is important for the development of the retraction pocket, as we can see the presence of retraction in children with normal function of the Eustachian tube. Therefore, prevention of inflammation is important in the treatment strategy of the retraction pocket.

## Data Availability

Not applicable.
